# Lentinan Improves Sodium Arsenite-Induced Hepatic Lipid Accumulation and Ferroptosis: Role of AMPK Signaling Pathway

**DOI:** 10.3390/toxics14080701

**Published:** 2026-08-07

**Authors:** Shunli Luo, Yekang Deng, Yan Lu, Yuan Yang

**Affiliations:** 1 Guangxi Key Laboratory of Environmental Exposomics and Entire Lifecycle Health, College of Public Health, Guilin Medical University, Guilin 541199, China; lsl@hnmu.edu.cn (S.L.);; 2 Department of Food Hygiene and Nutrition, College of Laboratory Medicine, Hunan University of Medicine, Huaihua 418000, China

**Keywords:** Lentinan, sodium arsenite, hepatic lipid accumulation, ferroptosis, AMPK signaling

## Abstract

Sodium arsenite (SA) is an environmental chemical toxin that induces hepatotoxicity and cellular ferroptosis under exposure. Ferroptosis is characterized by elevated ferrous ion levels and decreased glutathione peroxidase 4 (GPX4) activity. Lentinan (LNT), a bioactive polysaccharide derived from shiitake mushrooms, was investigated for its regulatory effects on SA-induced hepatotoxicity. In vivo experiments demonstrated that LNT ameliorated SA-induced hepatic lipid accumulation, increased GPX4 content and the mRNA levels of AMP-activated protein kinase (AMPK), and decreased ferrous ion concentrations in the livers of mice. However, co-administration of the AMPK inhibitor compound C and LNT negated these protective effects on hepatic lipid accumulation and ferroptosis-related markers. Complementary in vitro studies revealed that LNT activates AMPK signaling, mimicking the antagonistic effects of the AMPK agonist metformin (Met) on lipid accumulation and ferroptosis in hepatocytes exposed to SA. Notably, immunoblotting analyses indicated that treatment with LNT or Met increased the LC3-II/LC3-I ratio and upregulated the expression of ULK1 and the lipophagy receptor oxysterol-binding protein-related protein 8 (ORP8). Furthermore, co-immunoprecipitation assays demonstrated enhanced interaction between ORP8 and ULK1, suggesting activation of the AMPK-mediated ORP8/ULK1 signaling pathway. This pathway appears to play a protective role against SA-induced hepatic lipid accumulation and ferroptosis. Collectively, these findings elucidate the beneficial effects of LNT-mediated AMPK activation in mitigating SA-induced lipid accumulation and ferroptosis in the liver.

## 1. Introduction

Inorganic arsenic (iAs) is classified by the International Agency for Research on Cancer (IARC) and the World Health Organization (WHO) as a Group 1 carcinogen and is recognized as a major environmental contaminant of global concern. Both natural geological processes and human activities have led to unsafe arsenic levels in drinking water and food, affecting approximately 200 million people across more than 70 countries [1]. Among the various forms of iAs, sodium arsenite (SA, NaAsO_2_) is the most common and toxic trivalent species encountered in human exposure, making it a clinically significant toxicant rather than merely a laboratory chemical [2]. When ingested through contaminated water or food, iAs is absorbed in the gastrointestinal tract and primarily metabolized by the liver, which is the main organ where arsenic accumulates and exerts toxicity [2]. Epidemiological studies have demonstrated that environmental arsenic exposure significantly increases the risk of metabolic dysfunction-associated steatotic liver disease (MASLD) [3] and type 2 diabetes mellitus (T2DM) [4] in humans. Laboratory investigations have also shown that mitochondria are the primary targets of SA-induced liver toxicity [5]. Mechanistically, SA enters hepatocytes and strongly binds to sulfhydryl (-SH) groups in glutathione (GSH) and mitochondrial proteins, leading to GSH depletion, weakened antioxidant defenses, and excessive production of reactive oxygen species (ROS) [5,6]. Additionally, SA exposure causes mitochondrial swelling, loss of mitochondrial membrane potential (ΔΨm), mitochondrial oxidative stress, and impaired aerobic respiration in hepatocytes [5]. Furthermore, mitochondrial damage initiates ferritinophagy and the associated cellular ferroptosis by releasing mitochondrial reactive oxygen species (mtROS) and mitochondrial ferritin (FTMT) [7]. Previous research has shown that prolonged SA exposure enhances ferritinophagy-driven ferroptosis in chickens, which is linked to activation of the mammalian target of the rapamycin (mTOR)/uncoordinated 51-like kinase 1 (ULK1) autophagy signaling pathway [8]. Another study demonstrated that SA induces oxidative stress within cells [9], where excessive ROS interact with polyunsaturated fatty acids in cellular lipid membranes, causing lipid peroxidation and triggering ferroptosis. This process is characterized by increased levels of ferrous ions (Fe^2+^) and reduced activity of glutathione peroxidase 4 (GPX4) [10]. GPX4 is regarded as a unique cellular defense molecule against ferroptosis, catalyzing the oxidation of GSH to glutathione disulfide (GSSG), thereby reducing free radicals and helping prevent ferroptosis [11]. GSH acts as a crucial ROS scavenger and a cofactor for GPX4; thus, decreased GPX4 activity or its direct degradation results in elevated iron-dependent ROS, promoting ferroptosis in cells [12].

Lentinan (LNT) is a bioactive polysaccharide derived from shiitake mushrooms. Its molecular backbone consists of β-(1→3)-D-glucopyranosyl units, with side chains composed of glucose polymers linked by β-(1→6) and β-(1→3) bonds. Recent in vivo studies have demonstrated that LNT enhances autophagic flux via the adenosine 5′-monophosphate (AMP)-activated protein kinase (AMPK)/mTOR signaling pathway, accelerating the clearance of myelin debris by Schwann cells and promoting neurological recovery [13]. Our recent research has shown that LNT protects against liver damage and inflammation induced by SA [14]. Furthermore, LNT supplementation reduces hepatic steatosis caused by a high-fat diet (HFD) in mice [15]. Mechanistically, ferroptosis not only accelerates hepatocyte death but also leads to the accumulation of lipid peroxidation products that inhibit key enzymes responsible for fatty acid oxidation. This inhibition prevents free fatty acids (FFAs) from entering mitochondria for β-oxidation, causing their conversion into triglycerides (TG) within hepatocytes—a process linked to the development of alcoholic liver injury and MASLD [16]. AMPK, a critical intracellular energy sensor, acts as a key upstream regulator of cellular autophagy or lipophagy—a selective form of autophagy targeting lipid droplets, resulting in their ubiquitination and subsequent degradation [17]. Metformin (Met), a well-known AMPK activator, has been shown to stimulate AMPK activation and lipophagy in hepatocytes, thereby alleviating hepatic steatosis [18]. In this study, Met was used as a pharmacological positive control to validate the role of AMPK signaling in our SA-exposed hepatocyte model. Using this as a reference, we aimed to investigate whether LNT, as a natural polysaccharide, could similarly activate AMPK signaling to counteract SA-induced hepatic steatosis and ferroptosis.

## 2. Materials and Methods

### 2.1. Animal Experiment Protocol

Specific pathogen-free (SPF) male C57BL/6J mice, aged 9 to 10 weeks (w), were obtained from Hunan Slack Company, Changsha, China (SCXK (Xiang) 2021-0002). They were housed in an environment maintained at 20–24 °C with 40–60% humidity and a 12 h light/dark cycle. The experimental procedures were approved by the Animal Ethics Committee of Guilin Medical University (28 February 2024; GLMC20243129) and were strictly conducted in accordance with the 3R principles of experimental animal use and ethical guidelines for humane animal care. Sodium arsenite (NaAsO_2_, SA, CAS.7784-46-5, Sigma-Aldrich, St. Louis, MO, USA), as the toxic exposure agent (dissolved in deionized water), and Lentinan (LNT, CAS.37339-90-5, Shanghai Duma Biotechnology Co., Ltd., Shanghai, China) or an AMPK inhibitor compound C (CC, CAS.866405-64-3, ab120843, Abcam, Cambridge, UK), as the intervention agent, were dissolved in phosphate-buffered saline (PBS). Mice were randomized into four groups based on the principle of similar body weights (with a difference of less than 1 g); the sample size was set as 6 in each of the four groups, namely control (mice fed under conventional conditions with free access to drinking water); SA exposure (SA administered by gavage at a dose of 5.0 mg/kg body weight (bw) every other day for 6 w); LNT + SA (LNT administered by gavage at 50.0 mg/kg bw, followed 12 h later by SA gavage at 5.0 mg/kg bw every other day for 6 w); and CC + LNT + SA (CC 10 μg/g bw were administered intraperitoneally (i.p.) 30 min after the gavage of LNT at a dose of 50.0 mg/kg bw, followed 12 h later by SA gavage at 5.0 mg/kg bw every other day for 6 w), according to Lu et al.’s study [19]. At the end of the experiment, mice were fasted for 24 h, then anesthetized and euthanized. Serum and liver samples were collected for subsequent analyses.

### 2.2. Oil Red O Staining of Liver Tissue

Frozen liver samples were prepared using a Cryotime FSE frozen slicer (Thermo Fisher Scientific, Waltham, MA, USA) and placed in a cryostat. Tissue sections of 5 μm thickness were made using an OCT embedding medium and then stained with Oil Red O. The lipid distribution within the liver tissue was examined under an optical microscope (DMB5-2231P1, Motic Company, Xiamen, China). Fat deposits appeared as orange-red particles or clusters, while nuclei were stained blue after counterstaining. Semi-quantitative analysis of lipid accumulation was conducted by capturing microscope images.

### 2.3. Biochemical or Enzyme-Linked Immunosorbent Assay (ELISA) Procedures

Mouse liver tissue was collected and rinsed with the PBS buffer to remove residual blood. A liver homogenate was prepared by mixing the tissue with pre-chilled physiological saline at a ratio of 1 g of tissue to 9 mL of saline (10% *w*/*v*) using a Dounce homogenizer (Wheaton; Millville, NJ, USA) in an ice bath. The RPMI medium containing 0.05% type II collagenase was then added, and the mixture was incubated at 37 °C for 30 min. After repeated freeze–thaw cycles to fully lyse hepatocytes, the homogenate was centrifuged at 3056.3× *g* and 4 °C for 10 min. The supernatant was collected and kept on ice for further analysis.

A multifunctional microplate reader (Thermo Fisher Scientific, Waltham, MA, USA) was used to measure oxidative stress markers including malondialdehyde (MDA, X6743), superoxide dismutase (SOD, X6899), catalase (CAT, X6641), triglycerides (TG, DM-X6707), and total cholesterol (TC, DM-X6798), which were all sourced from Duma Biotechnology Co., Ltd, Shanghai, China. Additionally, GPX4 (X6703, Duma Biotech, Shanghai, China), total iron (Fe, DM-X6704, Duma Biotech, Shanghai, China), and ferrous ions (Fe^2+^, BC5415, Solarbio Biotech, Beijing, China) were measured following the ELISA kit protocols. Optical density (OD) readings were converted to concentrations (μmol/mL or nmol/mL) based on standard curves provided by the ELISA kits, and values were normalized per gram (g) of wet tissue and expressed as μmol/g tissue or nmol/g tissue.

Mouse serum samples were collected and centrifuged at 2414.9× *g* and 4 °C for 10 min. The serum supernatant was used to assess the levels of amino transaminase (ALT, C009-2, Jiancheng Biotech, Nanjing, China) or aspartate transaminase (AST, C010-2, Jiancheng Biotech, Nanjing, China) by biochemical analysis methods. OD values were measured with an ELISA reader according to the manufacturer’s instructions. These OD values were converted to Carmen units using a standard curve, and then further converted to units per liter (U/L) using the following formula: Carmen unit × 0.482 × dilution multiple.

### 2.4. Measurement of AMPK/ULK1 mRNA Levels in the Liver

Total mRNA was extracted from liver samples using the following procedure: The frozen liver tissue was placed into a 1.5 mL centrifuge tube. Then, 1 mL of the Trizol reagent was added. The tissue was thoroughly ground with a tissue grinder (Wheaton; Millville, NJ, USA) while kept in an ice water bath to create a homogenate. Next, 200 µL of chloroform was added and the mixture was shaken until it developed a pink, ice cream-like appearance. The sample was then incubated on ice for 10 min, followed by centrifugation at 15,093× *g* and 4 °C for 15 min. The supernatant was collected and mixed with an equal volume of isopropanol. After thorough mixing by inversion, the mixture was left to stand for 10 min. The supernatant was removed by centrifuging the sample twice, leaving a white precipitate which represented the tissue mRNA. The concentration and purity of the extracted mRNA were measured, with concentrations ranging from 200 to 2000 ng/μL and purity ratios (OD260/OD230) between 1.8 and 2.2. Reverse transcription reagents were then added according to the protocol to convert the mRNA into cDNA. The cDNA templates were combined in appropriate proportions for PCR amplification using primers specific for AMPK and ULK1 genes. A real-time quantitative polymerase chain reaction (RT-qPCR) was performed using an Applied Biosystems instrument (Thermo Fisher Scientific, Foster City, CA, USA). The relative mRNA levels were analyzed using the 2^−ΔΔCT^ method [20]. The forward and reverse primer sequences for the target genes were as follows: AMPK (forward: 5′-GTGACCAATGGTGTTCGTGC-3′; reverse: 3′-CACGCCCTTTCTCATCCACT-5′) and ULK1 (forward: 5′-AGAGGGCTGTGTACCGAGAT-3′; reverse: 3′-TCCTAGAGAGAACAGGGGGC-5′).

### 2.5. AML12 Hepatocyte Experiment Protocol

Frozen AML12 hepatocytes were thawed and recovered in a cell culture incubator (ESCO, Singapore). Cells were plated into cell culture flasks containing Dulbecco’s Modified Eagle Medium (DMEM) and cultured at 37 °C with 5% CO_2_. For the selection of SA exposure dose in the present experiments, that is, AML12 hepatocytes exposed to SA for 48 h at different dosages, the final concentrations of SA exposure were 0.0, 2.0, 4.0, and 8.0 μmol/L. Cell viability was assessed using the CCK-8 assay. Specifically, 10 μL of the CCK-8 solution was added to each well and incubated at 37 °C for 4 h. Absorbance (A) was then measured at 450 nm using a multifunctional microplate reader. Cell viability was calculated using the following formula: [(A of experimental well − A of blank well)/(A of control well − A of blank well)] × 100%. Each experimental dose was tested in triplicate.

Once the optimal SA concentration was identified, four groups were established to investigate the effects of LNT or AMPK signaling modulation: the control (hepatocytes were cultured normally in DMEM for 48 h); the SA group (hepatocytes were exposed to 4.0 μmol/L SA for 48 h); the LNT intervention (the LNT + SA group; hepatocytes were exposed to SA 4.0 μmol/L for 6 h, followed by co-treatment with 25.0 μmol/L LNT for 42 h); and the AMPK agonist Met intervention according to Park J et al.’s study [20] (the Met + SA group; hepatocytes were exposed to 4.0 μmol/L SA for 6 h, then co-treated with 2.0 mmol/L Met for 42 h).

### 2.6. Lipid Droplet Fluorescence Staining and Lipid Content Analysis

A 100 μL suspension of cells was seeded into each well of a 6-well plate. SA exposure or LNT intervention was applied once cells reached 70% to 80% confluence. After treatment, 1.0 mL of cell fixative was added to each well and incubated for 20–30 min. The fixative was then removed, and the wells were rinsed 2–3 times with deionized water. Subsequently, 1.0 mL of a 60% BODIPY staining solution (C2053M, Beyotime Biotechnology Co., Ltd., Nanjing, China) was added to each well and incubated in the dark at room temperature for 20~30 s. Green fluorescence was excited at approximately 493/503 nm using a microplate reader, and fluorescence was observed with an inverted fluorescence microscope (Leica GmbH, Wetzlar, Germany). For the assay of lipid content, the adherent cell suspension was collected, and then mixed with ELISA reaction reagents according to the manual introductions, thus determining the levels of TG and FFAs in AML12 hepatocytes.

### 2.7. Western-Blotting (WB) Experiment

Hepatocytes were collected and lysed using a RIPA buffer containing a 1% protease inhibitor. Total protein was extracted from the cells, and protein concentration was measured quantitatively using a BCA assay kit. The cell lysates were then heated in a water bath at 95 °C for 10 min. Immunoblotting was conducted to assess the relative expression levels of the autophagosome marker LC3, GPX4, long-chain acyl-CoA synthetase 4 (ACSL4), phosphorylated AMPK (p-AMPK), phosphorylated ULK1 (p-ULK1), FTMT, and oxysterol-binding protein-related protein 8 (ORP8). The WB procedure included preparing sodium dodecyl sulfate polyacrylamide gel electrophoresis (SDS-PAGE) gels, loading protein samples, electrophoresis, membrane transfer, and blocking. The following primary antibodies were used: anti-LC3 I/II (PA1-16931, Invitrogen, Carlsbad, CA, USA), anti-GPX4 (ab231174, Abcam, Cambridge, UK), anti-ACSL4 (PA5-27137, Invitrogen, Carlsbad, USA), anti-phospho-AMPKα1/2 (ab133448, Abcam, Cambridge, UK), anti-AMPK (ab80039, Abcam, Cambridge, UK), anti-phospho-ULK1 (PA5-104556, Invitrogen, Carlsbad, USA), anti-FTMT (MA568234, Invitrogen, Carlsbad, USA), and anti-ORP8 (PA5-110065, Invitrogen, Carlsbad, USA). These antibodies were applied individually and incubated overnight at 4 °C. Next, enzyme-conjugated secondary antibodies were added and incubated at room temperature for 2 h. Detection and imaging were performed using an enhanced chemiluminescence (ECL) kit. The WB bands were scanned using an infrared laser imaging system (LI-COR 9120, LI-COR Biosciences, Inc., Lincoln, NE, USA) and analyzed by relative quantification of band integral absorbance (IA), with β-actin serving as the internal control (IA_0_).

### 2.8. Co-Immunoprecipitation (Co-IP) Analysis

Lysis, protein extraction, and protein concentration measurement of hepatocytes were carried out using the same methods as those for WB. A portion of the cell lysate was reserved as the input sample, while the remainder was incubated with primary antibodies (anti-ORP8 and anti-ULK1 (ab167139, Abcam, Cambridge, UK)) for Co-IP. Additionally, a negative control was incubated with isotype IgG. After antibody incubation, magnetic beads were added to the supernatant to precipitate and wash the complexes. A loading buffer was then added to samples from the experimental group, negative control, and input, followed by WB analysis. Relative quantification was performed by analyzing integral absorbance levels, with β-actin as the internal reference (IA_0_).

### 2.9. Statistical Analysis

Data analysis and statistical graphing were performed using SPSS version 28.0 and GraphPad Prism version 8.0. Experimental results are expressed as the mean (M) ± standard deviation (SD). All data were first tested for normality using the Shapiro–Wilk test. Then the differences between groups were assessed using one-way analysis of variance (ANOVA) followed by the least significant difference (LSD) test. A *p*-value less than 0.05 was considered statistically significant.

## 3. Results

### 3.1. Characteristics of Mouse Body Weight and Liver Coefficient

As shown in Table 1, by the end of the experiment, the overall weight of mice in each group exhibited an increasing trend, with no significant differences observed among the groups. Liver weight in the SA exposure group and the CC + LNT + SA group showed no significant difference (*p* > 0.05), but both were increased compared to the control group. Liver weight in the LNT + SA group was lower than that in the SA exposure group or the CC + LNT + SA group (*p* < 0.05). Similarly, the liver coefficient (liver weight/body weight) in the SA exposure group and the CC + LNT + SA group was significantly higher than that in the control group, while the liver coefficient in the LNT + SA group was lower than that in the SA exposure group or the CC + LNT + SA group (*p* < 0.05).

### 3.2. LNT Regulates Arsenic-Induced Hepatic Lipid Distribution and AMPK/ULK1 Signaling

**Figure 1:**
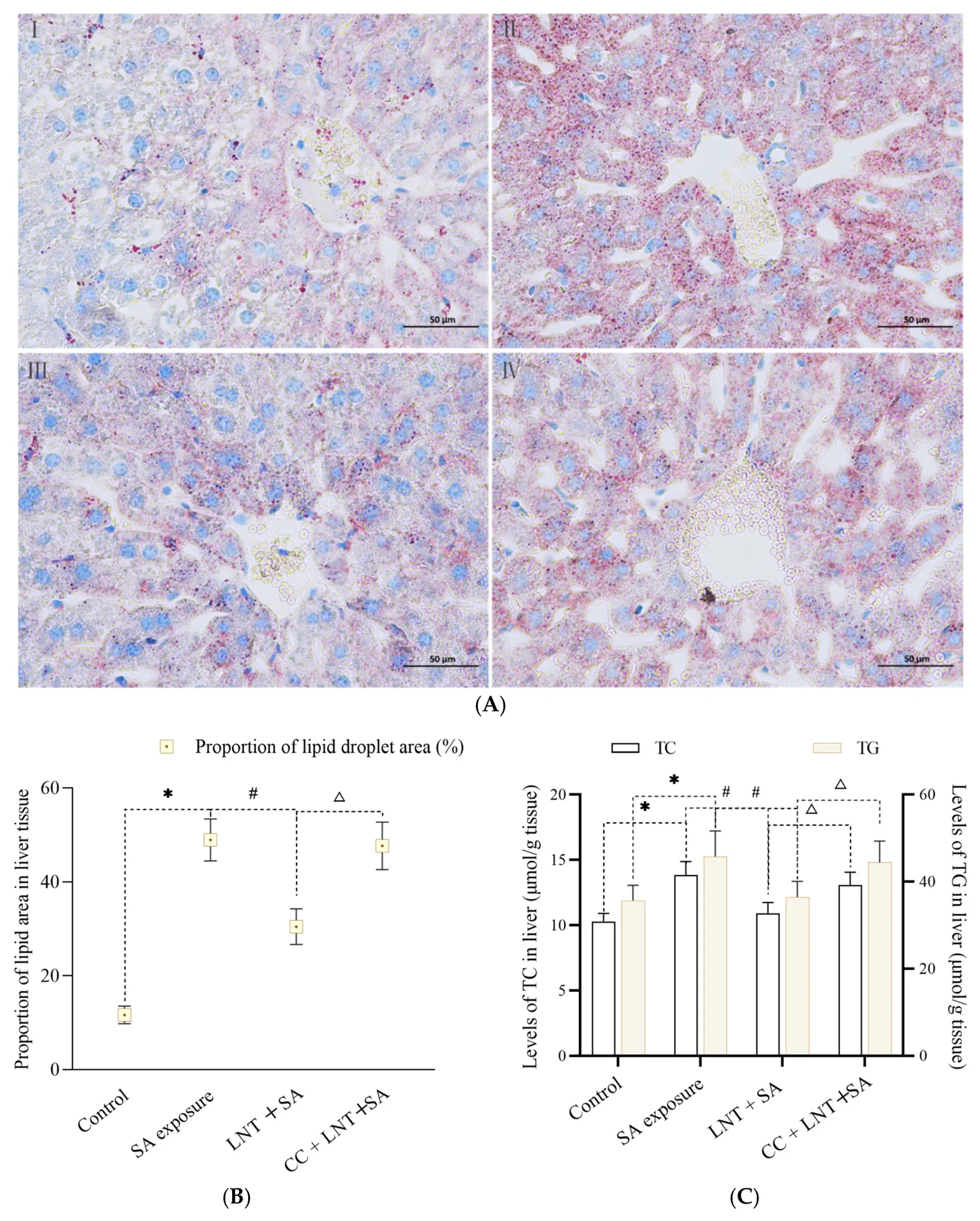

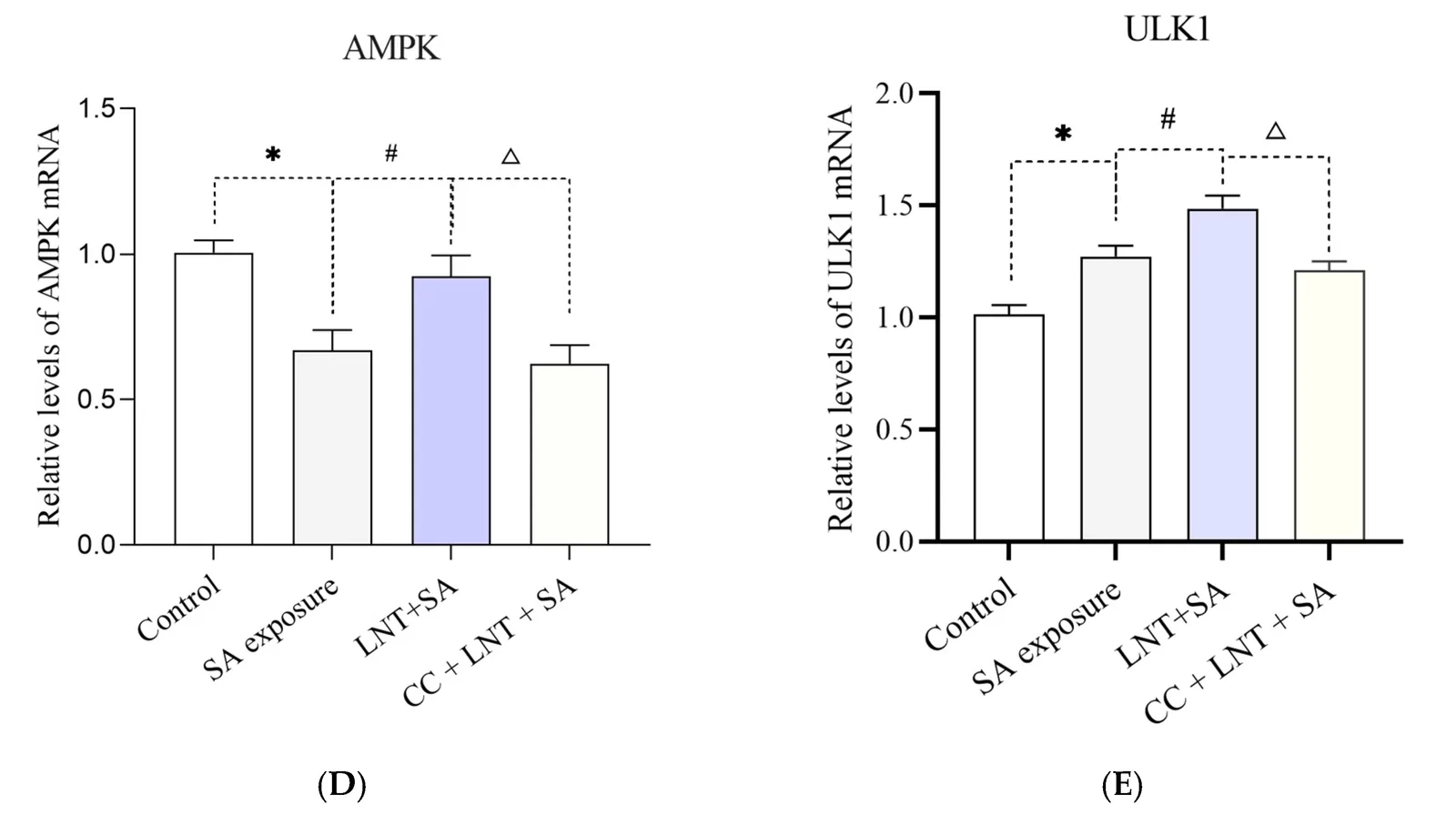
Characteristics of lipid distribution, lipid content, and AMPK/ULK1 signaling in the liver (Oil Red O staining, ×40). (**A**) I, control; II, SA exposure; III, LNT + SA; IV, CC + LNT + SA. SA exposure: 5.0 mg/kg Bw; LNT intervention: 50 mg/kg Bw. (**B**) Semi-quantitative evaluation of lipid distribution (%); (**C**) levels of TG and TC in the liver; (**D**,**E**) relative mRNA levels of AMPK and ULK1 in the liver. Data are expressed as the M ± SD, n = 6; *: compared with the control, *p* < 0.05; ^#^: compared with the SA exposure group, *p* < 0.05; ^△^: compared with the LNT + SA group, *p* < 0.05.

As shown in Figure 1A, in the control group, the cellular structure of liver tissue was clearer, with fewer fat particles and no obvious red lipid droplets, indicating low lipid content in the liver tissue. In the SA exposure group and the group treated with both the AMPK inhibitor and LNT (CC + LNT + SA group), the liver cells exhibited enhanced staining, and clustered red lipid droplets appeared around the cells, indicating elevated lipid accumulation in hepatic tissue. In contrast, the distribution of red lipid droplets appeared reduced in the LNT + SA group. Similarly, the semi-quantitative analysis of lipid droplets showed a significantly increased proportion of lipid area in the SA exposure group compared to that in the control, while the proportion of lipid area was downregulated in the LNT + SA group. Interestingly, the proportion of lipid area in the CC + LNT + SA group showed a significant rebound compared to that in the LNT + SA group (*p* < 0.05), resembling the characteristics observed in the SA exposure group, as shown in Figure 1B.

As shown in Figure 1C, the SA exposure group exhibited elevated levels of TG and TC compared to the control group. LNT intervention (LNT + SA) resulted in decreased levels of TG and TC compared to the SA exposure group. However, the combined intervention with the AMPK inhibitor CC and LNT against SA exposure (CC + LNT + SA) showed increased levels of TG and TC compared to the LNT + SA group (*p* < 0.05). As shown in Figure 1D,E, the SA exposure group demonstrated decreased AMPK and increased ULK1 mRNA levels compared to the control. The LNT intervention (LNT + SA) consistently increased the mRNA levels of AMPK and ULK1 compared to the SA exposure group, whereas the combined intervention with CC and LNT (CC + LNT + SA) resulted in a consistent downregulation of AMPK and ULK1 compared to the LNT + SA group (*p* < 0.05), as detailed in Table S1. These results indicate that LNT ameliorates SA-induced lipid accumulation in the liver and activates AMPK/ULK1 signaling, while inhibition of AMPK signaling significantly counteracts the beneficial effects of LNT on hepatic lipid distribution.

### 3.3. AMPK Inhibitor Regulates SA-Induced Liver Injury, Oxidative Stress, and GPX4 or Fe^2+^ Levels

**Figure 2:**
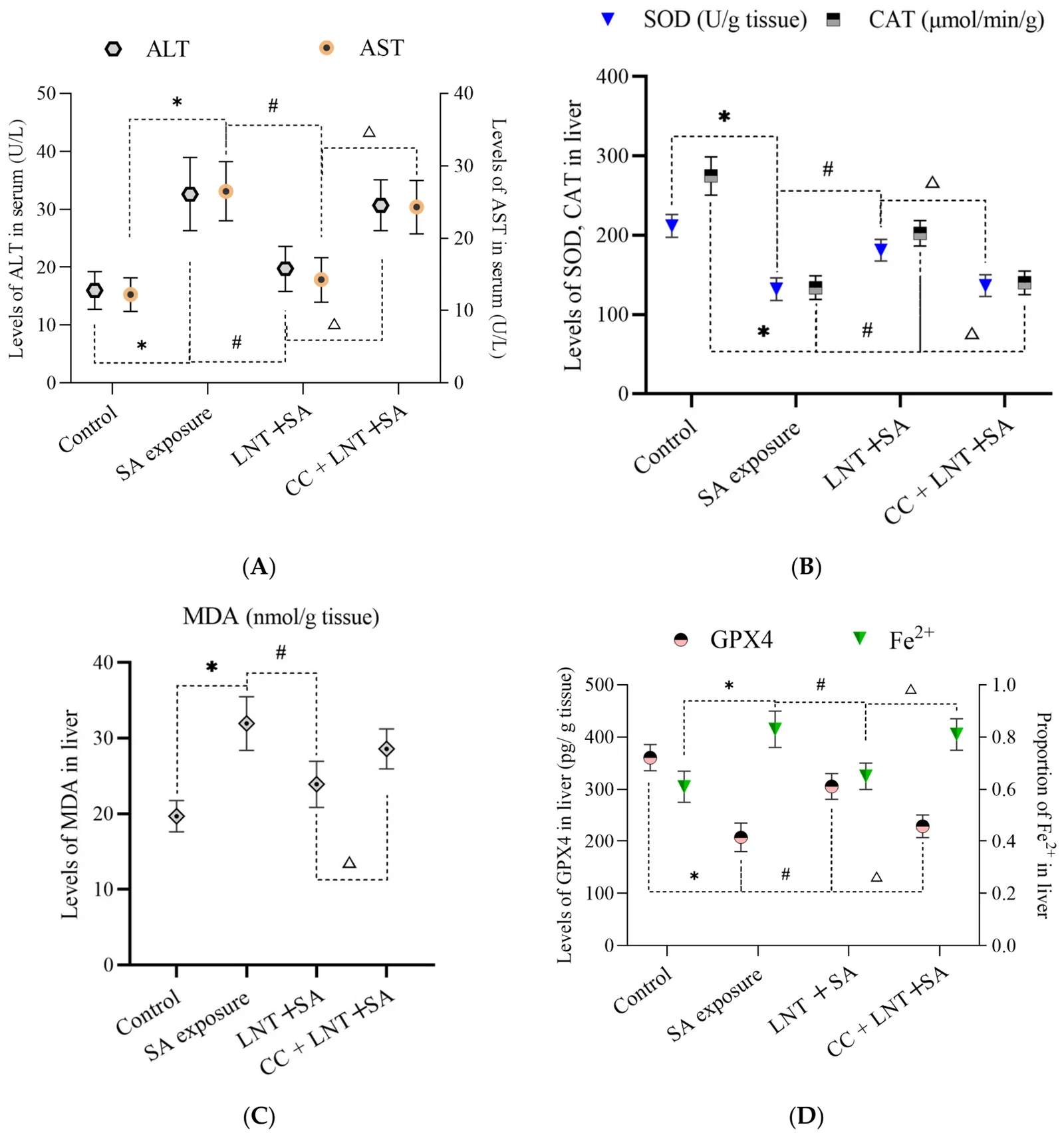
Characteristics of oxidative stress and ferroptosis markers. (**A**–**C**) Characteristics of ALT, AST, SOD, CAT, and MDA; (**D**) characteristics of GPX4 and the proportion of Fe^2+^ in the liver. Data are expressed as the M ± SD, n = 6; *: compared with the control, *p* < 0.05; ^#^: compared with the SA exposure group, *p* < 0.05; ^△^: compared with the LNT + SA group, *p* < 0.05.

As shown in Figure 2A–D, compared with the control group, the SA exposure group exhibited elevated levels of liver injury markers (ALT and AST), the lipid peroxidation product MDA, and Fe^2+^, while the levels of antioxidant enzymes (SOD, CAT, and GPX4) decreased in the liver (*p* < 0.05). Compared with the SA exposure group, LNT intervention (LNT + SA) significantly antagonized the changes in ALT, AST, MDA, Fe^2+^, SOD, CAT, and GPX4. However, combined treatment with the AMPK inhibitor CC and LNT significantly counteracted the LNT-mediated regulatory effects on liver injury, oxidative stress, GPX4, and Fe^2+^ in SA-exposed mice (*p* < 0.05), as detailed in Table S1. These results suggest that LNT ameliorates SA-induced hepatic damage, oxidative stress, and ferroptosis, while the addition of the AMPK inhibitor negates the beneficial effects of LNT on SA-induced hepatotoxicity.

### 3.4. Screening of LNT Intervention Dose and Evaluation of Lipid Content in Hepatocytes

**Figure 3:**
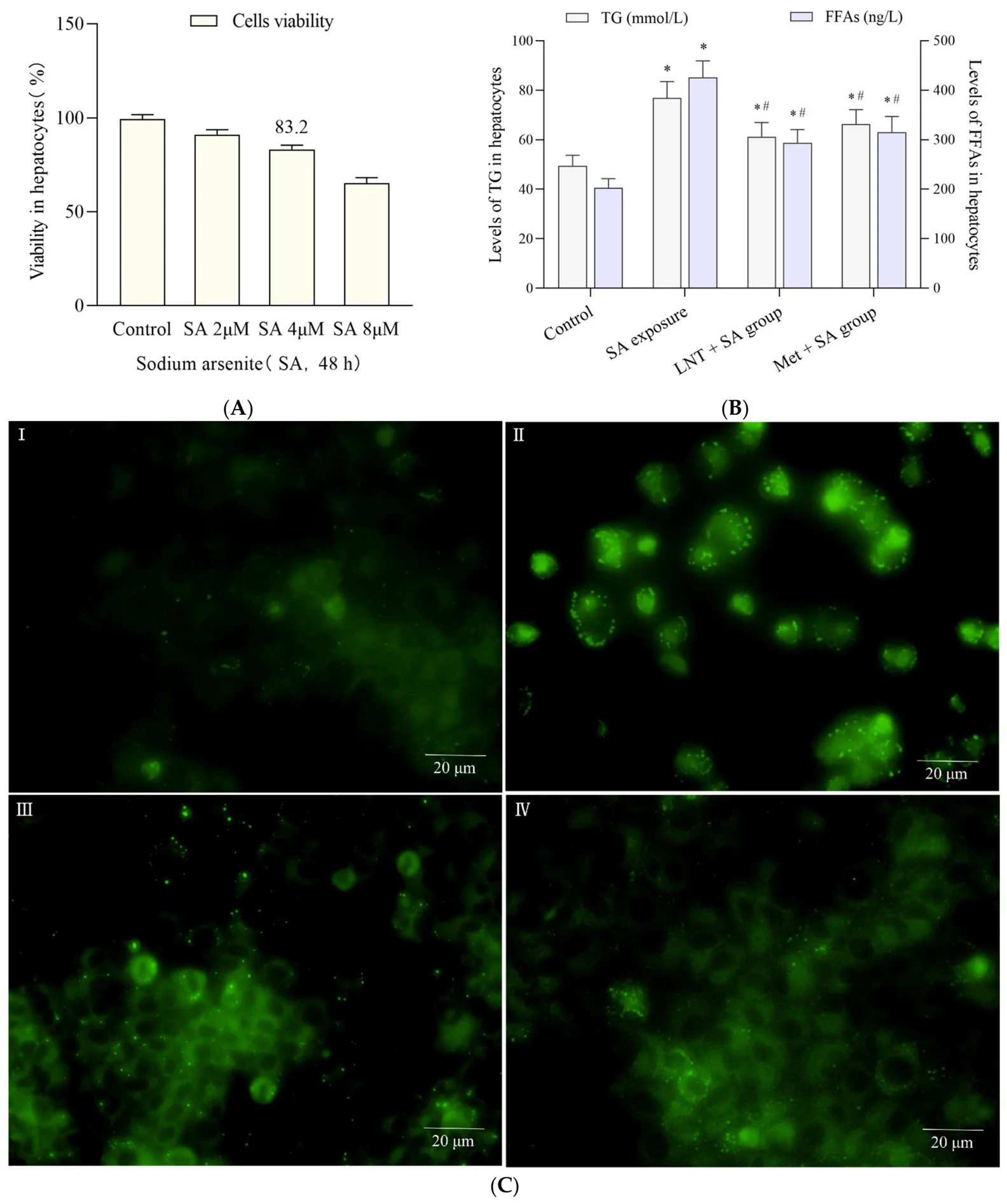
Characteristics of CCK-8 and lipid content in hepatocytes. (**A**) Viability in SA-exposed AML12 hepatocytes; (**B**) levels of TG and FFAs in hepatocytes; (**C**) characteristics of lipid droplet fluorescence staining in AML12 hepatocytes (I, control; II, SA exposure; III, LNT + SA group; IV, Met + SA group; magnification (×40); green fluorescence represents lipid particles). M ± SD, n = 3; *: compared with the control, *p* < 0.05; ^#^: compared with SA exposure, *p* < 0.05.

As shown in Figure 3A, the viability of AML12 hepatocytes exposed to SA was determined using the CCK-8 assay. The final concentrations of SA were 0.0, 2.0, 4.0, and 8.0 μmol/L. The CCK-8 assay showed that the average viability of AML12 hepatocytes decreased with increasing SA concentrations, with values of 99.3%, 91.0%, 83.2%, and 65.2%. The 4.0 μmol/L SA group, which had an average survival rate of 83.1%, was selected for subsequent AML12 hepatocyte experiments.

As shown in Figure 3B, the lipid content results exhibited similar characteristics, with elevated levels of TG and FFAs observed in the SA exposure group. Intervention with LNT (LNT + SA group) or Met (Met + SA group) significantly reduced the levels of TG and FFAs (*p* < 0.05), as detailed in Table S2.

As shown in Figure 3C, after 48 h of SA exposure in AML12 hepatocytes, lipid droplet fluorescence staining revealed increased green fluorescence intensity, with lipids appearing as particles or clumps inside or around the cells. Compared to the SA exposure group, the LNT intervention (LNT + SA group) or the AMPK agonist Met intervention (Met + SA group) showed reduced green fluorescence intensity, indicating an improvement in lipid accumulation within hepatocytes. The green fluorescence in the control group was weak, indicating few lipid droplets inside the cells. These results suggest that the LNT intervention or the activation of AMPK signaling ameliorates SA-induced lipid accumulation in hepatocytes.

### 3.5. LNT or the AMPK Activator Enhances Autophagy and Inhibits Ferroptosis in SA-Exposed Hepatocytes

**Figure 4:**
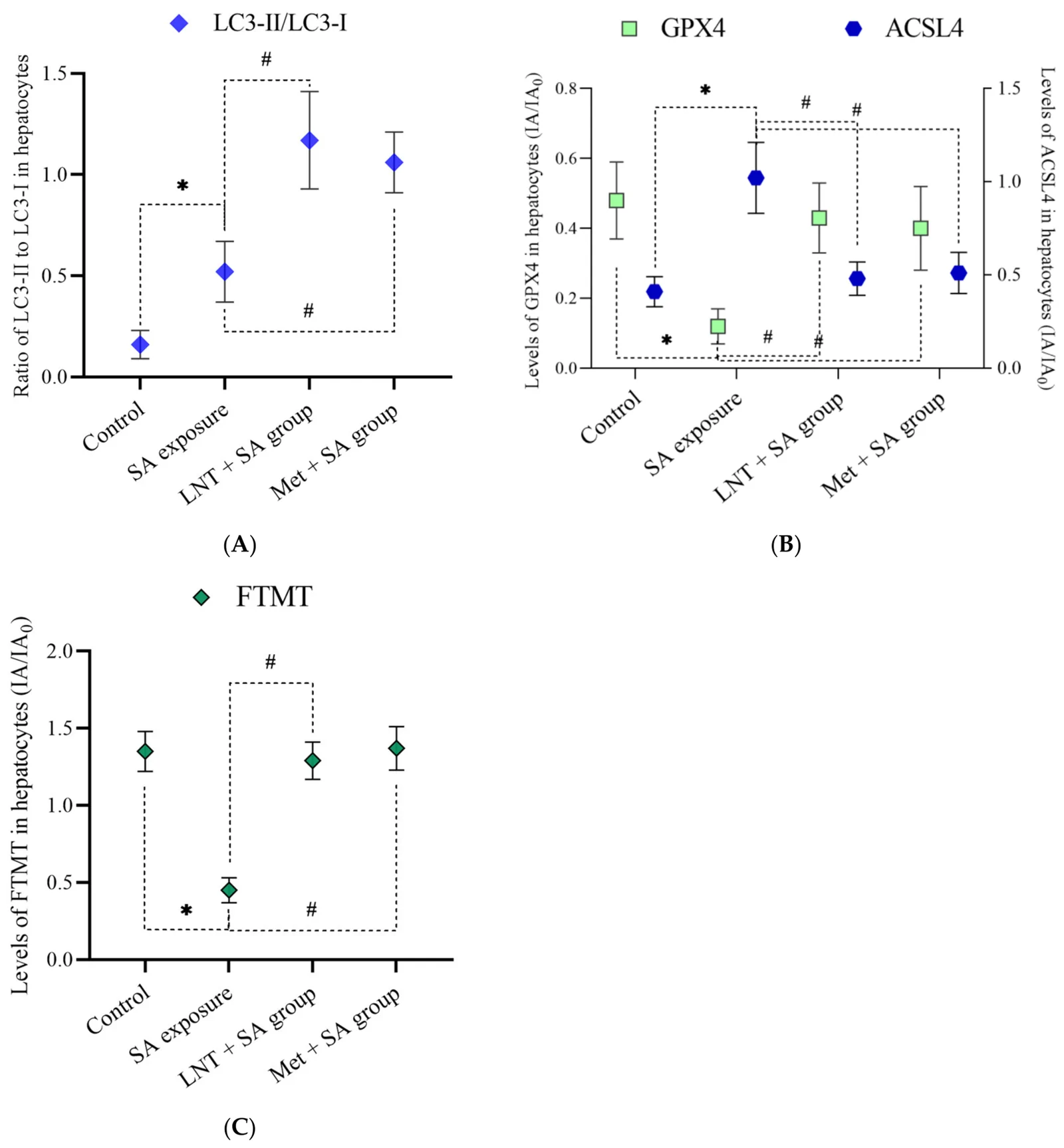
Characteristics of ferroptosis-related indices in hepatocytes. (**A**) Levels of the ratio of LC3-II to LC3-I in AML12 hepatocytes; (**B**) relative levels of GPX4 and ACSL4 in AML12 hepatocytes; (**C**) relative levels of FTMT in AML12 hepatocytes; n = 3, M ± SD; *: compared with the control, *p* < 0.05; ^#^: compared with SA exposure, *p* < 0.05.

As shown in Figure 4A–C, compared with the control group, SA exposure resulted in a significant increase in the ratio of LC3-II to LC3-I and an increase in the ferroptosis biomarker ACSL4, along with decreased levels of GPX4 and FTMT in hepatocytes (*p* < 0.05). Compared with the SA exposure group, intervention with LNT or the AMPK agonist Met consistently increased the ratio of LC3-II to LC3-I and upregulated the levels of GPX4 and FTMT while downregulating ACSL4 expression (*p* < 0.05). These results suggest that LNT or AMPK activators induce autophagic flux and alleviate SA-induced ferroptosis in AML12 hepatocytes, as detailed in Table S3 or Figures S1–S3.

### 3.6. LNT or the AMPK Agonist Activates ORP8 and ULK1 Signaling in SA-Exposed Hepatocytes

**Figure 5:**
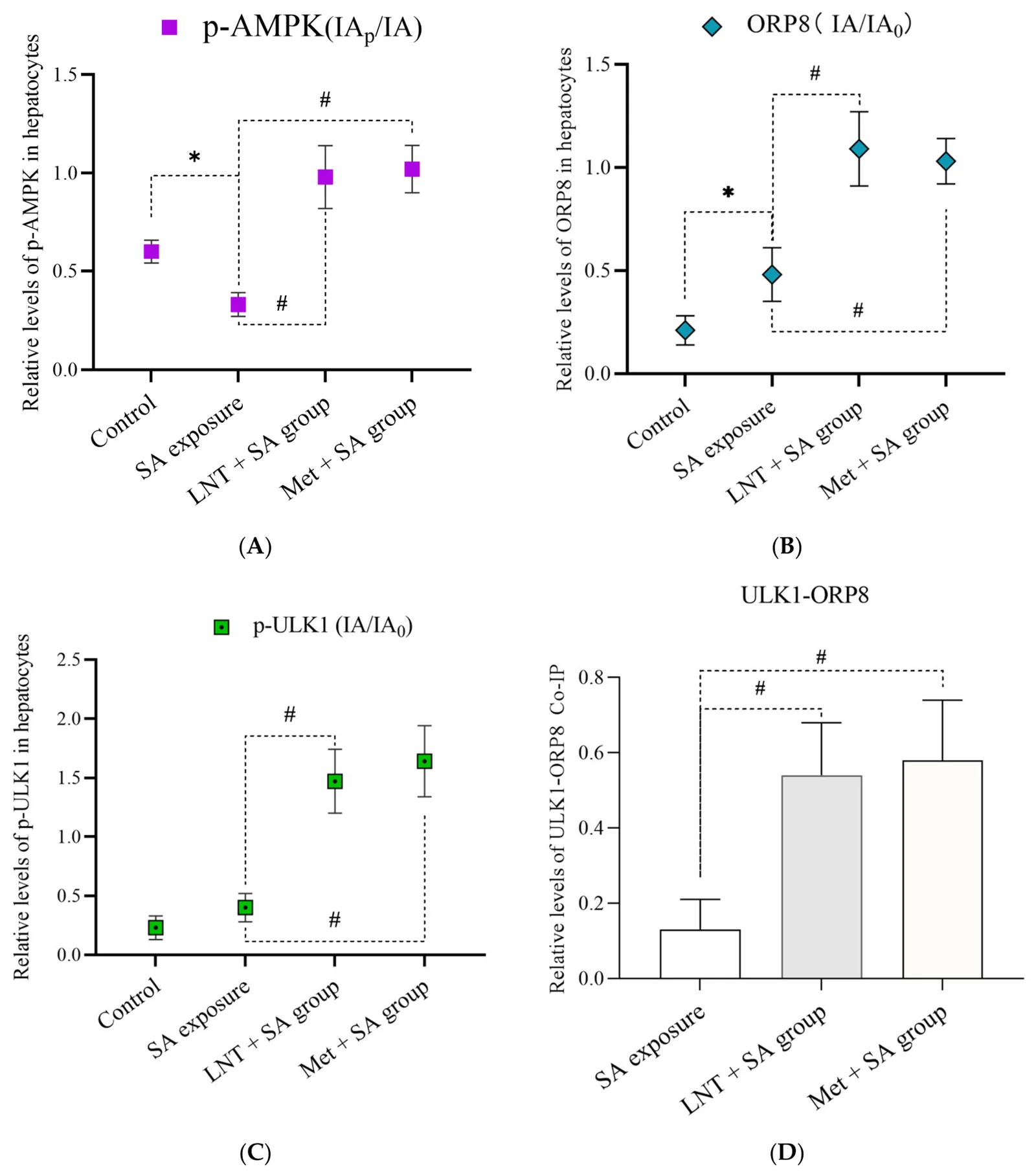

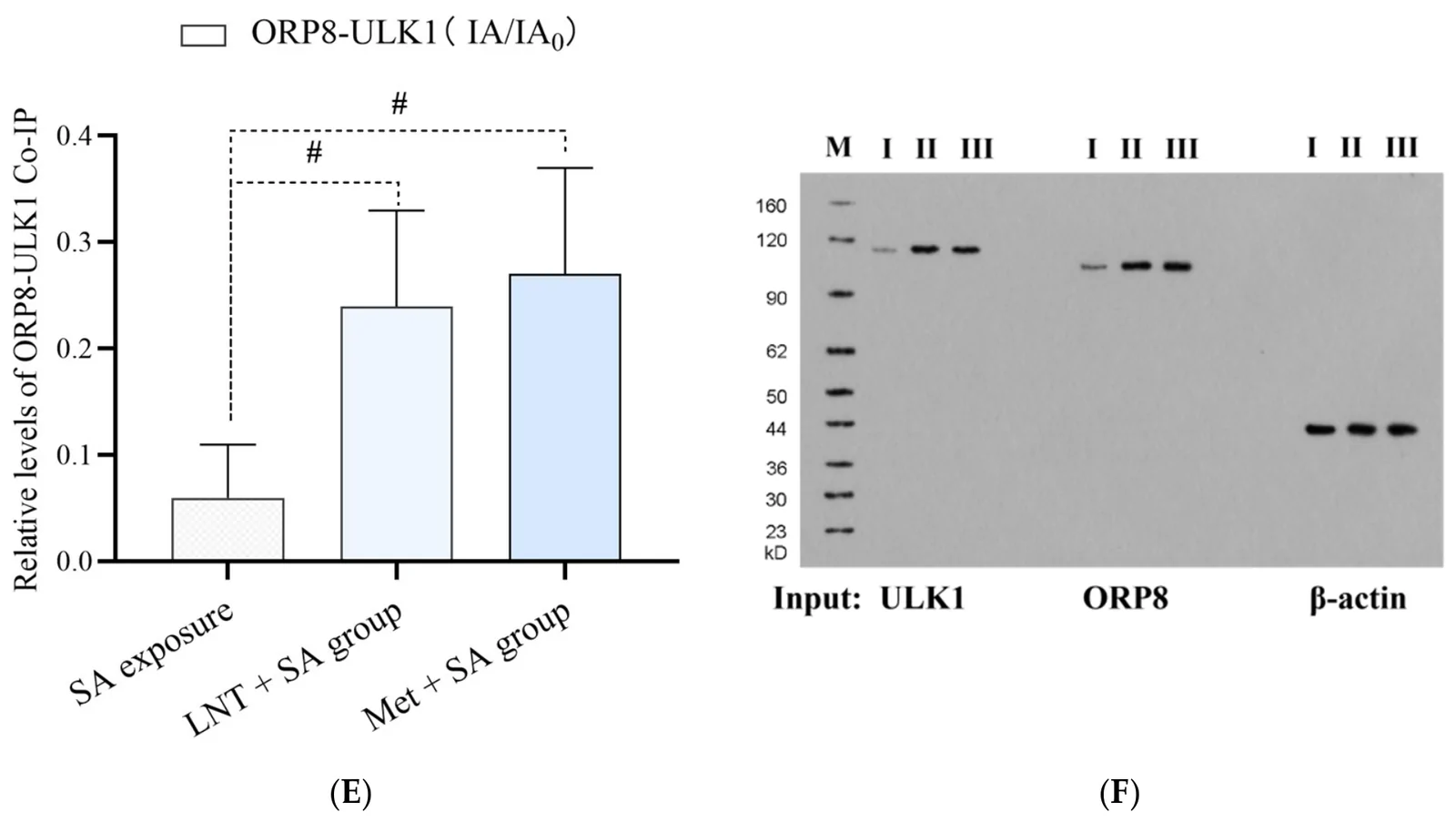
Characteristics of p-AMPK, ORP8, p-ULK1, and ORP8-ULK1 co-expression in hepatocytes. (**A**–**C**) Relative levels of p-AMPK, ORP8, or p-ULK1; (**D**,**E**) relative levels of ULK1-ORP8 or ORP8-ULK1 co-expression; (**F**) representative immunoprecipitation figures of ORP8 and ULK1. I: SA exposure; II: LNT + SA group; III: Met + SA group. n = 3, M ± SD; *: compared with the control, *p* < 0.05; ^#^: compared with SA exposure, *p* < 0.05.

As shown in Figure 5A–C, compared with the control group, the SA exposure group exhibited decreased levels of p-AMPK and increased levels of ORP8, while the change in p-ULK1 levels was not statistically significant (*p* < 0.05). Compared with the SA exposure group, intervention with LNT (LNT + SA group) or metformin (Met + SA group) resulted in a significant increase in the levels of p-AMPK, ORP8, and p-ULK1 (*p* < 0.05). These results suggest that the LNT intervention activates AMPK signaling and results in enhanced ORP8 and p-ULK1 signaling, which may contribute to the amelioration of lipid accumulation in SA-exposed AML12 hepatocytes.

ORP8, a bioactive lipophagy receptor-like protein, plays a key role in mediating lipophagy signaling and is regulated by AMPK signaling [21]. ULK1, the mammalian homolog of yeast autophagy-related gene 1 (ATG1), is essential for autophagy induction and can be activated by phosphorylation via AMPK signaling [22]. Here, compared to the SA exposure group, the immunoprecipitation results showed that the LNT or Met intervention resulted in a significant increase in the levels of ORP8-ULK1 co-expression (*p* < 0.05), as shown in Figure 5D–F. The above results suggest that LNT or the activation of AMPK signaling mediated the binding of ORP8-ULK1 signaling and consequently induced the activation of the autophagy or lipophagy signaling pathway in SA-exposed AML12 hepatocytes, as detailed in Table S3 or Figures S2–S4.

## 4. Discussion

Currently, ferroptosis is recognized as a toxicopathological hallmark associated with exposure to non-ferrous metals and metalloids, including arsenic, cadmium, and copper. Previous studies have demonstrated that SA induces hepatic mitochondrial dysfunction, the release of mtROS, and the activation of ferritinophagy [9], highlighting ferritinophagy-mediated ferroptosis in chicken hepatocytes [8]. Mitochondria are the primary sites of lipid metabolism within cells; mitochondrial dysfunction leads to the accumulation of superoxide anion radicals (O_2_^−^) and mitochondrial metabolic substrates such as lipids, contributing to lipid accumulation in cells [23]. Furthermore, hepatic lipid accumulation triggers lipotoxicity and induces MASLD by mediating endoplasmic reticulum stress, oxidative stress, and ferroptosis [24]. Intracellular Fe^2+^ is mainly stored in mitochondria in the form of FTMT. Due to mitochondrial dysfunction or damage, FTMT is released from mitochondria and binds to nuclear receptor coactivator 4 (NCOA4)—an autophagy-related protein that recruits the autophagosome protein LC3—to activate ferritinophagy via the lysosomal pathway, leading to intracellular Fe^2+^ overload and subsequent ferroptosis [25,26]. In addition to Fe^2+^ overload, decreased GPX4 levels are also considered a hallmark of ferroptosis in cells [11]. Mechanistically, inhibition of GPX4 activity in mitochondria contributes to ferroptosis by exacerbating lipid peroxidation and mitochondrial dysfunction [27]. Furthermore, GPX4 activity can be regulated by AMPK signaling, a crucial upstream pathway governing cellular autophagy and lipophagy [17,28]. Recent studies have revealed a protective role of activated hepatic AMPK signaling in the progression of MASLD [29]. AMPK signaling upregulates GPX4 expression, which antagonizes ferroptosis [30]. Additionally, the bioactive form of AMPK inhibits lipid synthesis by phosphorylating and suppressing acetyl-CoA carboxylase (ACC), which alleviates the inhibition of mitochondrial carnitine palmitoyltransferase-1 (CPT-1) activity, facilitating the transport of long-chain fatty acyl-CoA into mitochondria for fatty acid oxidation [31]. Our recent investigations demonstrated that SA exposure induces hepatic oxidative stress and lipid accumulation in mice [32]. Complementary studies have shown that SA exposure impairs AMPK/PGC-1α signaling-mediated mitochondrial biogenesis in animal livers, resulting in disrupted mitophagy and hepatic steatosis [33]. Furthermore, oleic acid activates AMPK and increases nuclear FoxO1 localization, thereby attenuating arsenic-induced cardiomyocyte hypertrophy [34]. Prior research has also indicated that treatment with LNT promotes autophagic flux via activation of the AMPK signaling pathway [13], whereas dysfunction of AMPK-mediated autophagy contributes to MASLD progression [35].

In the present study, we found that LNT antagonizes SA-induced hepatic lipid accumulation and lipid peroxidation, as evidenced by the reduced lipid area proportion, TG, and TC, along with alleviated hepatic oxidative stress. Additionally, the LNT intervention regulated ferroptosis-related indices, which are characterized by decreased Fe^2+^ levels. Interestingly, combined treatment with the AMPK inhibitor CC and LNT reversed the beneficial effects of LNT on SA-induced hepatic lipid accumulation, lipid peroxidation, and ferroptosis-related indices, resembling the changes observed in SA-exposed mice. These findings suggest that LNT attenuates SA-induced hepatic lipid accumulation and lipid peroxidation in mice, thereby improving Fe^2+^ overload and consequently ferroptosis. However, inhibition of AMPK signaling counteracts the regulatory effects of LNT on SA-induced lipid accumulation, oxidative stress, and ferroptosis in the liver. LNT is a bioactive polysaccharide predominantly composed of a β-1,6;1,3-glucan structure and exhibits potent antioxidant activity [13]. Previous studies have shown that LNT upregulates the expression of peroxisome proliferator-activated receptor α (PPARα)—a downstream target of the AMPK pathway in the mouse liver—which promotes mitochondrial fatty acid oxidation and ameliorates hepatic lipid accumulation [36]. PPARα also activates AMPK signaling [37]. Another study demonstrated that LNT induces phosphorylation and activation of AMPK signaling to clear myelin debris in glial cells, thereby alleviating sciatic nerve injury [13]. A pertinent question arises regarding the mechanism by which orally administered LNT, a high-molecular-weight β-glucan largely resistant to mammalian digestive enzymes, reaches the liver to modulate AMPK signaling. We propose two convergent pathways underlying LNT-mediated regulation of AMPK signaling. First, pharmacokinetic studies have confirmed that orally administered LNT achieved maximal plasma concentrations within one hour in rodent models, and LNT-treated Caco-2 cell monolayer model assays indicated that clathrin-mediated endocytosis and macropinocytosis participate in LNT transport and absorption [38]. Our in vitro data further corroborate that LNT directly activates AMPK signaling in hepatocytes, supporting its capacity as a direct AMPK modulator, which depends on its bioavailability. Second, LNT serves as a fermentable substrate for gut microbiota; the unabsorbed LNT fraction reaches the colon, where microbial fermentation generates bioactive metabolites such as short-chain fatty acids (SCFAs) [39]. These metabolites enter the portal circulation and are established activators of hepatic AMPK signaling [40].

AMPK functions as an intracellular energy sensor regulated by adenosine monophosphate (AMP) and adenosine triphosphate (ATP), serving as a crucial upstream signaling molecule that governs cellular autophagy and lipophagy [17]. ULK1, a mammalian autophagy-initiating kinase, is phosphorylated and activated by AMPK, triggering cellular autophagy in a model of LPS-induced macrophage inflammation [41]. Additionally, phosphorylation of the hepatic AMPK/ULK1 signaling pathway indicates lipophagy activation in the liver, contributing to the improvement in MASLD [29]. A previous study demonstrated that ULK1, which acts downstream of AMPK, induces the initiation of lipophagy in hepatocytes [42]. The AMPK-ULK1 signaling pathway is considered an upstream mechanism for activating lipophagy in adipocytes [43]. In our in vitro experiments, Met was utilized strictly as a positive control to confirm that the AMPK–lipophagy axis is functionally operative in SA-exposed hepatocytes. Notably, the LNT treatment recapitulated the effects of Met, including the alleviation of lipid accumulation, the restoration of ferroptosis markers (GPX4, ACSL4, and FTMT), and the upregulation of lipophagy-associated signals (p-AMPK, p-ULK1, ORP8, and the LC3-II/I ratio). Specifically, ORP8 is a bioactive protein that regulates lipophagy by localizing on lipid droplets and acting as a regulatory factor mediating this process. Furthermore, ORP8-mediated lipophagy is associated with AMPK signaling, and activated AMPK directly interacts with ORP8, which is its enzymatic substrate, leading to phosphorylation and activation of ORP8 [21]. ORP8 is located at the membrane contact site (MCS) between the endoplasmic reticulum (ER) and mitochondria, where it transports phosphatidylserine (PS) from the ER to mitochondria [44]. PS serves as a precursor for mitochondrial phosphatidylethanolamine (PE) synthesis. It has been found that ORP8 regulates mitochondrial respiratory function by interacting with the mitochondrial outer membrane protein tyrosine phosphatase-interacting protein 51 (PTPIP51) through its functional lipid transport domain (ORD) [44]. In the present study, LNT intervention resulted in elevated ORP8 levels in SA-exposed hepatocytes, suggesting a significant role for ORP8 in regulating mitochondrial function and lipophagy. Moreover, both LNT and Met enhanced the co-expression of ORP8 and ULK1, indicating activation of ORP8/ULK1-dependent lipophagy. Collectively, these findings suggest that LNT acts as a natural AMPK activator engaging the downstream ORP8/ULK1-associated lipophagy pathway, similar to the classical AMPK agonist Met, underscoring the translational potential of LNT as a botanical modulator of AMPK signaling in arsenic-induced hepatotoxicity. Additionally, several studies have elucidated that the AMPK signaling pathway regulates ferroptosis and lipid metabolism. For instance, AMPK activation upregulates the expression of GPX4, a key ferroptosis enzyme, thereby inhibiting ferroptosis in hippocampal neurons of diabetic mice [30]. Liraglutide activates AMPK signaling and suppresses ferroptosis, leading to improvements in lipid metabolism in a T2DM-associated MASLD model induced by a high-fat diet combined with streptozotocin injection [45]. In the present in vivo study, LNT intervention upregulated the mRNA levels of AMPK/ULK1 signaling components, suggesting that AMPK activation is associated with amelioration of SA-induced hepatic lipid accumulation, oxidative stress, and ferroptosis.

In summary, our experiments demonstrate that LNT counteracts hepatic lipid accumulation and ferroptosis in SA-exposed mice and AML12 hepatocytes. These effects are associated with activation of the AMPK-mediated signaling pathway. Notably, the interaction between ORP8 and ULK1 may represent a downstream signaling mechanism mediated by AMPK that initiates lipophagy, thereby contributing to the alleviation of hepatic lipid accumulation and the improvement in ferroptosis. However, due to inherent target heterogeneity and pathway crosstalk within the intracellular ferroptosis signaling network, the relationship between ferroptosis and lipid metabolism remains incompletely understood. Consequently, this study has limitations that warrant further investigation. First, the regulatory role of LNT in AMPK signaling during arsenic-induced ferroptosis requires systematic elucidation in future studies. Moreover, given the complexity of the AMPK-mediated lipophagy signaling pathway, subsequent research should explore the therapeutic potential of targeting LNT-mediated AMPK signaling and establish lipophagy or ferroptosis intervention models to clarify the mechanisms by which LNT mitigates arsenic-induced hepatic steatosis and ferroptosis disorders in murine models. Methodologically, the ELISA-based GPX4 measurements employed herein quantify protein abundance rather than intrinsic catalytic activity. Considering that SA, as a trivalent arsenical, avidly binds protein thiols and may induce post-translational modifications that suppress enzymatic function, classical biochemical activity assays will be essential to confirm GPX4 peroxidase activity in future investigations.

## 5. Conclusions

This study is the first to demonstrate that LNT activates the AMPK-mediated ORP8/ULK1 signaling pathway, which plays a role in mitigating SA-induced hepatic lipid accumulation and ferroptosis. These results offer novel mechanistic insights into the pathogenesis of hepatotoxicity associated with heavy metal exposure and suggest potential therapeutic targets for future intervention approaches.

## Figures and Tables

**Table 1:** Mouse weight, liver weight, and relative liver weight in each group.

Group	Control	SA Exposure	LNT + SA	CC + LNT + SA
Weight (g)	24.28 ± 0.50	24.27 ± 0.82	24.22 ± 0.70	24.30 ± 0.65
Liver weight (g)	1.03 ± 0.08	1.39 ± 0.07 *	1.16 ± 0.09 ^#^	1.41 ± 0.06 *^,△^
Relative liver weight (g/g, %)	4.22 ± 0.49	5.71 ± 0.37 *	4.67 ± 0.28 ^#^	5.75 ± 0.34 *^,△^

Data were expressed as M ± SD, *n* = 6; SA exposure group, SA 5.0 mg/kg bw; LNT intervention, 50 mg/kg bw; CC intervention: 10 μg/g bw. *: compared with the control, *p* < 0.05; ^#^: compared with the SA exposure group, *p* < 0.05; ^△^: compared with the LNT + SA group, *p* < 0.05.

## Data Availability

All data generated in this study are included in the article/Supplementary Materials. Further inquiries can be directed to the corresponding author.

## Supplementary Materials

The following supporting information can be downloaded at https://www.mdpi.com/article/10.3390/toxics14080701/s1, Table S1. Characteristics of liver injury, oxidative stress, lipid content, or ferroptosis indices; Table S2. LNT or the AMPK agonist downregulates lipid content in SA-exposed hepatocytes; Table S3. Immunoblot analysis of ferroptosis- and lipophagy-related markers in SA-exposed hepatocytes. Figure S1. Representative immunoblot images of LC3-II, LC3-I, GPX4, and ACSL4; Figure S2. Representative immunoblot images of ORP8 and p-ULK1; Figure S3. Representative immunoblot images of p-AMPK and FTMT; Figure S4. Representative immunoprecipitation images of ORP8 and ULK1.

## Abbreviations

The following abbreviations are used in this manuscript:

LNT	Lentinan	CC	Compound C
SA	Sodium arsenite	ATP	Adenosine triphosphate
iAs	Inorganic arsenic	AMPK	Adenosine 5′-monophosphate-activated protein kinase
IARC	International Agency for Research on Cancer	WHO	World Health Organization
MASLD	Metabolic dysfunction-associated steatotic liver disease	MASH	Metabolic dysfunction-associated steatohepatitis
T2DM	Type 2 diabetes mellitus	ACSL4	Long-chain acyl-CoA synthetase 4
TG	Triglyceride	NCOA4	Nuclear receptor coactivator 4
mtROS	Mitochondrial reactive oxygen species	L-ROS	Lipid reactive oxygen species
FTMT	Mitochondrial ferritin	PPARα	Peroxisome proliferator-activated receptor α
HFD	high-fat diet	Nrf2	Nuclear factor erythroid 2-related factor 2
mTOR	Mammalian target of rapamycin	LIR	LC3-interacting region
ULK1	Uncoordinated 51-like kinase 1	CE	Cholesteryl ester
ROS	Reactive oxygen species	RT-qPCR	Real-time quantitative Polymerase Chain Reaction
GSH	Glutathione	ELISA	Enzyme-linked immunosorbent assay
GSSG	Glutathione disulfide	FFAs	Free fatty acids
GPX4	Glutathione peroxidase 4	Fe^2+^	Ferrous ion
ANOVA	One-way analysis of variance	M	Mean
SD	Standard deviation	WB	Western blotting
LSD	Least significant difference	Co-IP	Co-immunoprecipitation
MDA	Malondialdehyde	IA	Integral absorbance
SOD	Superoxide dismutase	TC	Total cholesterol
CAT	Catalase	AMP	Adenosine monophosphate
SPF	Specific pathogen-free	A	Absorbance
O_2_^−^	Superoxide anion radicals	SDS-PAGE	Sodium dodecyl sulfate polyacrylamide gel electrophoresis
Met	Metformin		
