# Supplementary material for: Lentinan Improves Sodium Arsenite-Induced Hepatic Lipid Accumulation and Ferroptosis: Role of AMPK Signaling Pathway
Source: Toxics. 2026 Aug 7;14(8):701. doi: 10.3390/toxics14080701 (PMC13517948; doi:10.3390/toxics14080701)
Supplement: Supplementary file 1 [file toxics-14-00701-s001.zip › toxics-4443603-supplementary.pdf]

Supplementary materials for the article "Lentinan Improves Sodium Arsenite-Induced Hepatic Lipid Accumulation and Ferroptosis: Role of AMPK Signaling Pathway"

Table S1. Characteristics of liver injury, oxidative stress, lipid content, or ferroptosis indices

Indices	Groups			
	Control	SA exposure	LNT＋SA	CC+LNT＋SA
Proportion of lipid area (%)	11.62±1.87	48.91±4.47*	30.44±3.82#	47.67±5.04△
P values	—	5.73×10^{-13}	1.11×10^{-7}	3.20×10^{-7}
ALT (U/L)	15.96±3.25	32.65±7.60*	19.72±3.94#	30.74±4.38△
P values	—	1.45×10^{-5}	2.73×10^{-4}	1.25×10^{-3}
AST (U/L)	12.18±4.30	26.52±4.08*	14.25±5.11#	24.33±3.67△
P values	—	1.28×10^{-5}	8.39×10^{-5}	6.43×10^{-4}
SOD (U/g tissue)	211.69±16.32	132.07±11.15*	171.22±14.59#	138.55±13.71△
P values	—	4.41×10^{-9}	1.04×10^{-4}	6.68×10^{-4}
CAT (μmol/min/g)	274.74±24.16	133.99±12.97*	202.49±15.94#	140.06±14.83△
P values	—	9.39×10^{-12}	1.37×10^{-6}	4.93×10^{-6}
MDA(nmol/g tissue)	19.67±2.07	31.94±3.55*	22.90±4.05#	28.57±2.63△
P values	—	1.61×10^{-6}	7.98×10^{-5}	0.006
TC（μmol/g tissue）	10.29±0.61	13.84±1.02*	10.92±0.83#	13.08±0.98△
P values	—	8.16×10^{-7}	1.18×10^{-5}	3.72×10^{-4}
TG（μmol/g tissue）	35.68±3.50	45.76±5.91*	36.49±3.61#	44.45±4.87△
P values	—	1.10×10^{-3}	2.23×10^{-3}	6.90×10^{-3}
GPX4（pg/ g tissue）	360.59±24.98	207.58±27.95*	305.58±24.65#	229.51±21.67△
P values	—	1.11×10^{-9}	1.27×10^{-6}	3.55×10^{-5}
Total Fe content (μmol/ g tissue)	28.85±3.45	32.74±4.06	29.70±3.53	31.51±3.72
Proportion of Fe^{2+}	0.61±0.06	0.83±0.07*	0.65±0.05#	0.81±0.06△
P values	—	3.70×10^{-6}	4.74×10^{-5}	1.78×10^{-4}
AMPK mRNA	1.05±0.04	0.70±0.03*	0.92±0.07#	0.62±0.06△
P values	—	2.62×10^{-10}	4.98×10^{-7}	3.69×10^{-9}
ULK1 mRNA	1.01±0.04	1.27±0.05*	1.49±0.06#	1.21±0.04△
P values	—	9.93×10^{-9}	1.41×10^{-7}	2.88×10^{-9}

Data were showed as M±SD, n=6; LSD test was applied to evaluate the statistical difference among groups. *Compared with Control group, $P < 0.05$; #Compared with SA exposure group, $P < 0.05$; △Compared with LNT+SA group, $P < 0.05$.

Table S2. LNT or the AMPK agonist downregulates lipid content in SA-exposed hepatocytes

Indices	Groups			
	Control	SA exposure	LNT+SA	Met +SA
TG (mmol/L)	53.86±3.22	83.58±6.68*	66.56±5.73*#	69.96±6.75*#
P values	—	2.32×10^{-4}	*0.027; #0.007	*0.009; #0.020
FFAs (ng/L)	202.76±18.42	585.87±33.76*	401.77±26.60*#	415.40±31.52*#
P values	—	1.72×10^{-7}	*2.49×10^{-5}; #4.38×10^{-5}	*1.53×10^{-5}; #7.59×10^{-5}

Data were showed as M±SD, n=3; *Compared with Control group, $P < 0.05$; #Compared with SA exposure group, $P < 0.05$. Met, metformin. *P* values were showed as results of multiple comparison by LSD test.

Table S3. Immunoblot analysis of ferroptosis- and lipophagy-related markers in SA-exposed hepatocytes

Indices	Groups			
	Control	SA exposure	LNT+SA	Met + SA
GPX4 (IA/IA_0)	0.44±0.11	0.12±0.05*	0.41±0.10#	0.50±0.12#
P values	—	0.004	0.007	0.002
ACSL4 (IA/IA_0)	0.40±0.12	1.02±0.22*	0.44±0.12#	0.39±0.09#
P values	—	8.23×10^{-4}	0.001	7.43×10^{-4}
FTMT (IA/IA_0)	1.35±0.13	0.45±0.08*	1.29±0.12#	1.37±0.14#
P values	—	1.57×10^{-5}	2.60×10^{-5}	1.33×10^{-5}
LC3-II to LC3-I	0.16±0.07	0.52±0.15*	1.17±0.24#	1.06±0.15#
P values	—	0.028	0.001	0.004
p-AMPK (IA_p/IA)	0.60±0.06	0.33±0.05*	0.98±0.16#	1.02±0.20#
P values	—	0.048	4.97×10^{-4}	3.35×10^{-4}
p-ULK1 (IA/IA_0)	0.23±0.10	0.40±0.11	1.47±0.27#	1.64±0.30#
P values	—	0.361	2.92×10^{-4}	1.06×10^{-4}
ORP8 (IA/IA_0)	0.21±0.07	0.48±0.13*	1.09±0.18#	1.03±0.11#
P values	—	0.033	4.04×10^{-4}	7.92×10^{-4}
ORP8-ULK1 (IA/IA_0)		0.06±0.05	0.24±0.09#	0.27±0.10#
P values		—	0.037	0.021

ULK1-ORP8 (IA/IA_0)	0.13±0.08	0.54±0.14#	0.58±0.16#
P values	—	0.009	0.006

Data were showed as M±SD, n=3; *Compared with Control group, $P < 0.05$; #Compared with SA exposure group, $P < 0.05$. *P* values were showed as results of multiple comparison by LSD test.

Supplementary Figures

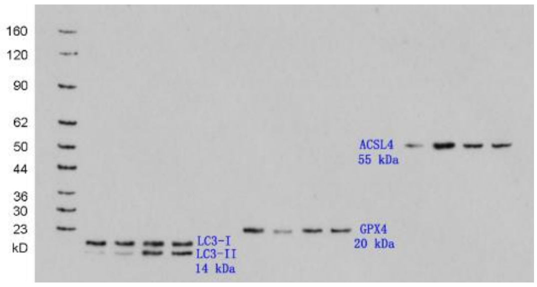

Figure S1. *Representative immunoblot images of LC3-II, LC3-I, GPX4, and ACSL4*

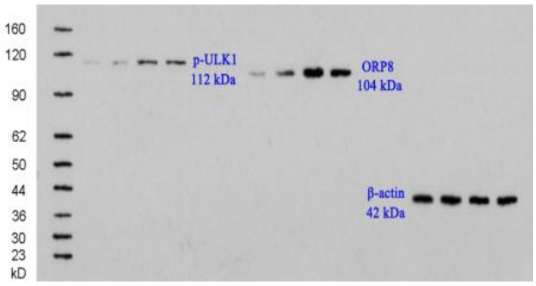

Figure S2. *Representative immunoblot images of ORP8 and p-ULK1*

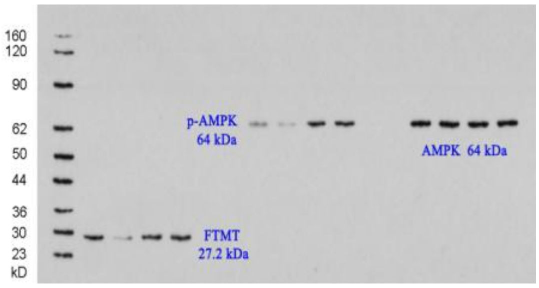

Figure S3. *Representative immunoblot images of p-AMPK and FTMT.*

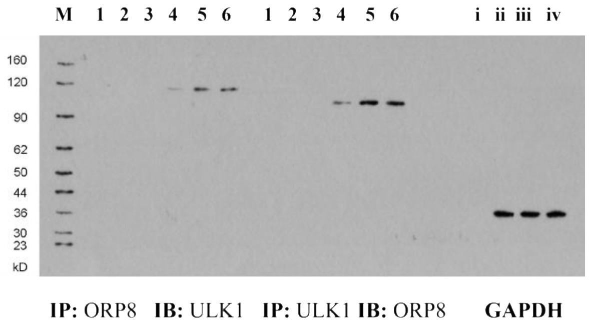

Figure S4. *Representative immunoprecipitation images of ORP8 and ULK1*

1~3: 4~6'IgG isoform control;

4: SA exposure; 5: rAPN + SA group; 6: Met + SA group;

i: GAPDH' IgG isoform control;

ii~iv: GAPDH internal reference.
